# Stent treatment improves cerebral microcirculatory disorder and blood–brain barrier function in internal carotid artery stenosis via intercellular adhesion molecule 1 modulation

**DOI:** 10.1002/ccs3.70058

**Published:** 2025-12-14

**Authors:** Kuo Li, Chuansuo Zhang, Li xuan Wang, Xiaoxuan Wang, Ruyue Wang

**Affiliations:** ^1^ No. 2 Department of Neurology Cangzhou Central Hospital Cangzhou China; ^2^ Radiointervention Department Cangzhou Central Hospital Cangzhou China; ^3^ Research Department Cangzhou Central Hospital Cangzhou China

**Keywords:** blood‐brain barrier, cerebral microcirculatory disorder, intercellular adhesion molecule 1, internal carotid artery stenosis, stent placement, watershed infarction

## Abstract

This study investigates the molecular mechanisms of early stent placement intervention in mitigating neurovascular unit damage and cerebral microcirculatory disorder (CMD) associated with severe internal carotid artery stenosis (ICAS). By utilizing a rat model of severe ICAS, early stent placement was found to improve cerebral blood flow, restore blood–brain barrier (BBB) integrity, and alleviate cognitive deficits by downregulating intercellular adhesion molecule 1 (ICAM1) expression. Transcriptomic analysis highlighted ICAM1's role in neurovascular repair by modulating inflammatory pathways and BBB‐associated tight junction proteins. In vitro experiments supported that ICAM1 knockdown enhanced BBB function by reducing inflammatory cytokines and promoting cell proliferation and migration. However, rescue experiments demonstrated that ICAM1 overexpression impeded the therapeutic effect of stent placement by exacerbating CMD and BBB disruption through upregulation of matrix metalloproteinase‐9 (MMP‐9) and inflammatory cytokines. These findings suggest that targeting ICAM1‐related pathways could optimize stent treatment strategies, emphasizing the importance of ICAM1 regulation in reducing the risk of watershed infarction and improving therapeutic outcomes in ICAS management.

## Graphical abstract

Transcriptomic analysis reveals the molecular mechanism by which early stent placement improves neurovascular unit damage and cerebral microcirculatory disorder induced by severe internal carotid artery stenosis through ICAM1 regulation.

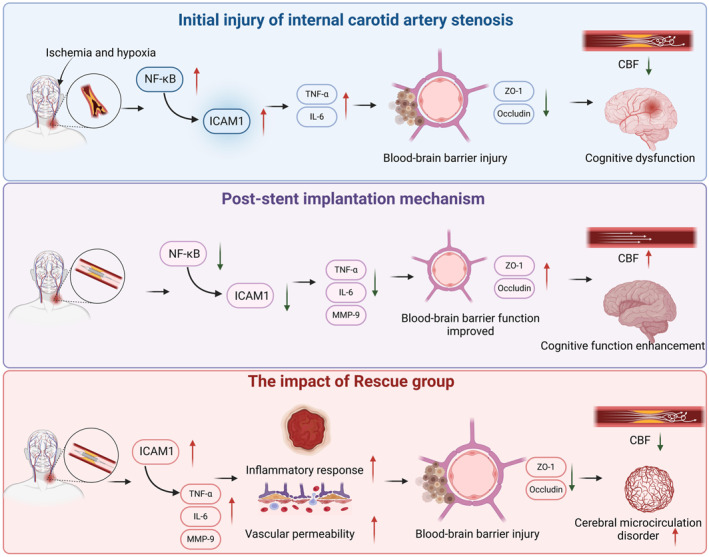

## 1 INTRODUCTION

Internal carotid artery stenosis (ICAS) is a common atherosclerotic vascular disease that severely affects cerebral blood flow (CBF) and has become a major cause of ischemic stroke. Pathophysiological studies indicate that ICAS leads to a decrease in cerebral perfusion pressure, resulting in disturbances in local cerebral microcirculation, and through exacerbating damage to the neurovascular unit, it induces blood–brain barrier (BBB) dysfunction.^1^, ^2^ BBB is located at the brain capillary wall, which is composed of endothelial cells. This multifactorial synergy increases the risk of watershed infarction and significantly impacts patient prognosis.^3^, ^4^ Clinically, stent implantation is a common treatment for ICAS, especially for improving CBF and reducing stroke risk.^5^ However, despite the widespread use of stent technology, the specific molecular mechanisms by which it influences cerebral microcirculation and BBB repair remain unclear.^6^, ^7^, ^8^ Previous studies have shown that stent implantation may alter hemodynamics, thereby compromising the integrity of the BBB and ultimately affecting its function.^6^ Notably, how to effectively restore BBB integrity while reducing inflammation remains a critical scientific challenge. Investigating the molecular regulatory mechanisms during stent treatment, particularly the role of inflammation‐related factors, will help optimize therapeutic strategies and minimize complications.

Intercellular adhesion molecule 1 (ICAM1) serves as an essential cell‐surface glycoprotein involved in mediating leukocyte–endothelial interactions and modulating vascular inflammatory responses. Studies have shown that under inflammatory stimuli, elevated ICAM1 expression promotes the migration and adhesion of inflammatory cells, thereby exacerbating vascular damage and tissue inflammation.^9^, ^10^, ^11^ ICAM1 influences blood–brain barrier (BBB) stability and permeability by modulating the expression levels of key tight junction components, including zonula occludens‐1 (ZO‐1) and occludin.^12^, ^13^, ^14^ Additionally, ICAM1 is overexpressed in various ischemic diseases, and its dysregulation is considered a key mechanism underlying neurovascular unit damage. Although previous studies have confirmed ICAM1's pivotal role in atherosclerosis and ischemic stroke,^15^, ^16^, ^17^, ^18^, ^19^ its specific function in ICAS‐induced cerebral microcirculatory disorder (CMD) and BBB disruption remains unclear. Notably, in the context of stent therapy, the dual role of ICAM1—potentially promoting BBB repair while exacerbating inflammation and undermining treatment efficacy—has yet to be fully elucidated.

Recent advances in bioinformatics and omics technologies have significantly enhanced the depth and scope of molecular mechanism studies. As a high‐throughput technology, transcriptomics can comprehensively reveal differentially expressed genes (DEGs) during disease progression, offering new insights through functional enrichment analysis and pathway predictions. Furthermore, the integration of various in vivo and in vitro experimental techniques, such as laser speckle contrast imaging (LSCI), immunohistochemistry, western blotting, and co‐culture technologies, provides robust support for validating the functions of candidate molecules. Current research studies indicate that animal models of ICAS, combined with early stent implantation interventions, allow for the evaluation of therapeutic effects and underlying mechanisms at both the systemic and molecular levels.^20^, ^21^ However, although previous studies have preliminarily explored the impact of stent therapy on CBF and the BBB, a detailed investigation into the regulatory mechanisms of key molecules remains lacking. Based on early literature and background research, we hypothesized that early stent placement could improve ICAS‐induced CMD and BBB dysfunction by modulating the ICAM1 signaling pathway.

**FIGURE 1:**
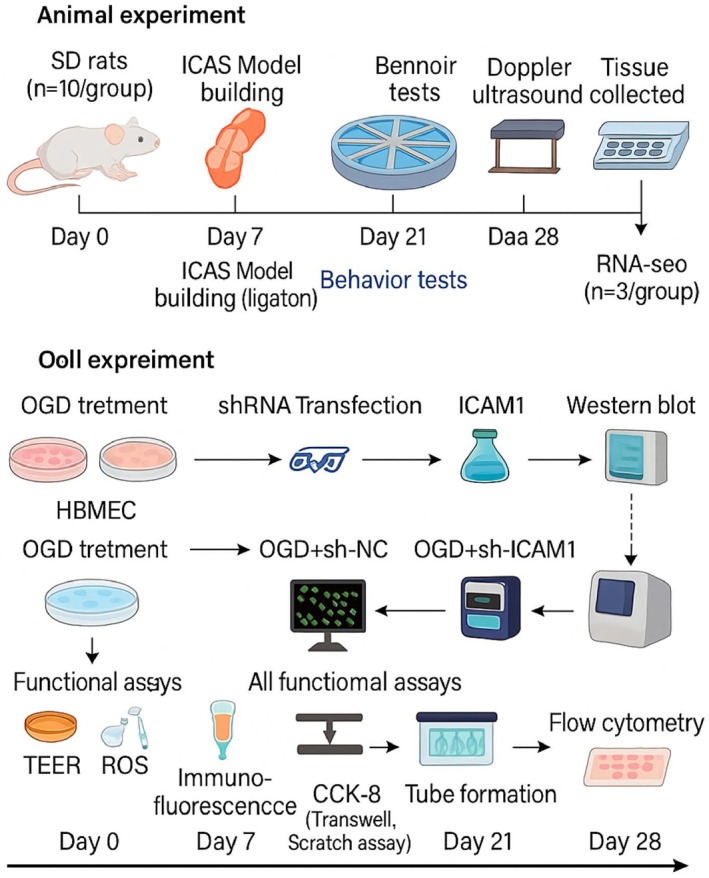
Schematic diagram of the experimental design.

Our study delves into clarifying how early stent implantation improves neurovascular unit damage and CMD induced by ICAS through ICAM1 modulation. Moreover, it explores the dual role of ICAM1 in inflammation and BBB repair. We systematically analyzed ICAM1‐related regulatory mechanisms through a combination of animal modeling, transcriptomic analysis, and in vitro validation experiments (Figure 1). The findings provide crucial theoretical support for optimizing stent therapy strategies, particularly in the context of targeting ICAM1‐related pathways to enhance therapeutic efficacy and reduce complication risks. The scientific value of this study lies in advancing the current understanding of ICAS and its therapeutic mechanisms, laying the foundation for personalized treatment plans. Clinically, it offers new directions for stroke prevention and treatment, particularly by improving BBB function and cerebral microcirculation, which may help reduce the incidence of watershed infarctions and enhance long‐term patient prognosis.

## 2 MATERIALS AND METHODS

### 2.1 Establishment of a severe ICAS rat model

**FIGURE 2:**
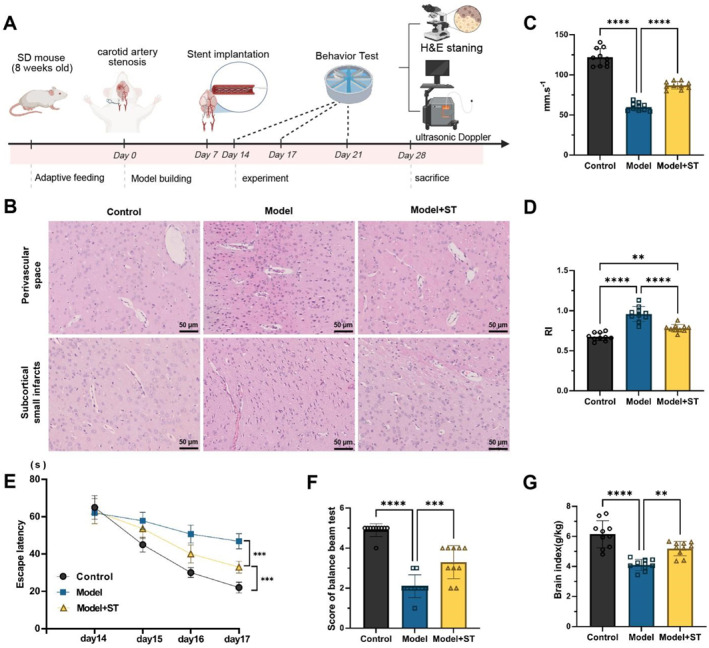
Experimental Design and Effectiveness Evaluation of Stent Implantation in Improving Severe ICAS. (A) Schematic of the experimental process, illustrating the modeling of unilateral severe ICAS, stent implantation, behavioral testing, and histological analysis. (B) H&E staining showing vascular structural changes at the site of ICAS. (C) Doppler ultrasound measurement of PSV in the ICA. (D) Doppler ultrasound measurement of the resistance index in the ICA. (E) Morris water maze test assessing learning and memory in rats. (F) Beam balance test evaluating motor coordination in rats. (G) Post‐mortem brain index assessing brain tissue volume changes in rats. Data are presented as mean ± standard error, with all experiments repeated three times and each group consisting of 10 mice. Statistical analysis was performed using ANOVA followed by Tukey's post‐hoc test. *p < 0.05, **p < 0.01, ***p < 0.001, ****p < 0.0001. ICAS, internal carotid artery stenosis.

To mimic severe unilateral ICAS in rats, we used 8‐week‐old male Sprague‐Dawley (SD) rats (200–250 g, Catalog No. 101, procured from Beijing Vital River Laboratory Animal Technology Co., Ltd.), which were acclimated for a week prior to the experiment. Rats in the control group underwent no ligation or stenosis surgery. Pentobarbital sodium (50 mg/kg, i.p.) was used to induce anesthesia, and then rats in the Model group (ligation only) and Model + ST group (ligation plus stent implantation) underwent surgery to induce unilateral ICAS by ligating the right internal carotid artery (ICA) with silk sutures. On day 7 post‐surgery, rats in the stent implantation group received nickel‐titanium alloy stents (diameter: 1.5 mm; wall thickness: 100 μm; length: 5 mm) through a minimally invasive procedure under the same anesthesia regimen to restore blood flow. Behavioral assessments (Morris water maze [MWM] and balance beam test) were conducted to evaluate cognitive function. On day 28, rats were euthanized, and brain tissues were immediately harvested and snap‐frozen in liquid nitrogen for subsequent analyses.^22^ The animal model and associated procedures are outlined in Figure 2A.

### 2.2 In vivo rescue experiment

A randomization procedure was selected to divide the Model + ST group into two subgroups, one of which received no further intervention, and the other was designated as the rescue group. Under deep anesthesia with intraperitoneal pentobarbital sodium (50 mg/kg), rats were secured in a stereotaxic apparatus. According to a standard rat brain atlas, the following stereotaxic coordinates were used: AP +1.2 mm, ML 2.6 mm (6° angle), DV 7.0 mm. A total of 2 μL of ICAM1‐overexpressing lentiviral vector (approx. 1E9 PFU, injection rate: 0.2 μL/min) was slowly injected into the target region utilizing a microsyringe. Following the injection, the needle was retained in place for 5 min prior to gradual withdrawal. The scalp was then sutured.^23^ Behavioral tests (MWM and balance beam test) were conducted post‐injection, and brain tissues were collected on day 28 for further analyses.^22^ The ICAM1‐overexpressing lentiviral vector was purchased from GeneChem Co., Ltd.

### 2.3 Doppler ultrasound imaging

High‐resolution ultrasound Doppler imaging (Vevo 3100, VisualSonics, FUJIFILM, Canada) was used to evaluate hemodynamic changes in the ICA after stent placement. A 20 MHz high‐frequency probe was used for real‐time imaging. Rats were lightly anesthetized utilizing 1.5%–2% isoflurane (R510‐22, RWD, China), fixed in the supine position, and the neck region was exposed. Ultrasound gel (Parker Laboratories, USA) was applied for signal transmission. Blood flow velocity (Peak Systolic Velocity, PSV) and Resistance Index (RI) were tested utilizing both color Doppler and pulsed‐wave Doppler modes. RI was calculated as RI = (PSV − EDV)/PSV, (EDV = end‐diastolic velocity). Measurements were taken at 3 weeks post‐surgery in the control, model, and model + ST groups. Data were analyzed using Vevo LAB software to assess the effects of stenting on vascular patency and hemodynamics.

### 2.4 MWM test

To assess learning and memory ability in rats, the MWM test was conducted on day 7 after stent placement. Over four successive days, each rat completed four daily training sessions in the water maze, with each session beginning from a distinct quadrant. The hidden platform was kept in a constant location throughout the training period. The duration required for the animal to locate the platform—termed escape latency—was measured in each trial. In cases where the platform was not located within 60 s, the rat was manually directed to the platform and allowed to rest on it for 10 s to promote spatial learning. On day 5, a probe test was performed to assess spatial learning. All sessions were video‐recorded and analyzed using software (Dongle Natural Gene Life Science Co., USA) to extract data such as swimming trajectory, speed, latency, and time spent in the target quadrant. The testing environment was kept quiet to ensure consistent results.

### 2.5 Balance beam test

To evaluate motor coordination and functional recovery, the balance beam test was employed. The setup featured a narrow beam (90 cm long, 1 cm wide) positioned between two square platforms (10 cm × 10 cm). In a quiet environment, each rat was placed on one platform for a 10‐s acclimation, then allowed to walk for 40 s. Motor performance was evaluated utilizing a 0–5 scoring system, considering walking ability, balance maintenance, and ability to reach the opposite platform. Two blinded evaluators independently scored each trial, and the average score was used for analysis. This test was utilized to examine the significance of stent placement on neurological and motor function recovery.

### 2.6 Hematoxylin and eosin (H&E) staining

To visualize the structural changes at the site of arterial stenosis, tissues were fixed in 4% paraformaldehyde (P6148, Sigma‐Aldrich, USA) for 24 h. Following fixation, specimens underwent sequential dehydration and clearing, followed by paraffin infiltration and embedding. Serial sections (5 μm thickness) were prepared utilizing a rotary microtome. The slides were then processed through xylene for deparaffinization and rehydrated using an ethanol gradient. Nuclear staining was achieved with hematoxylin (GHS132, Sigma‐Aldrich, USA) for 5 min. Subsequent differentiation was implemented utilizing 1% hydrochloric acid ethanol for 30 s, after which nuclear contrast was restored using ammonia water bluing. Cytoplasmic components were counterstained with eosin (HT110232, Sigma‐Aldrich, USA) for 2 min. Finally, the sections were dehydrated, cleared, and sealed with coverslips. Morphological observations and image capture were conducted using a Zeiss Axio Observer 7 microscope (Germany).

### 2.7 Brain organ index

To evaluate the relative brain size of mice as an indirect indicator of cognitive function, the brain organ index was calculated. Upon completion of the experimental procedures, rats were deeply anesthetized with pentobarbital sodium. The whole brain was then carefully extracted, and surface moisture was blotted using filter paper before weighing. The wet weight of the brain tissue (in grams) was recorded. The brain organ index was calculated as: Brain Organ Index = Brain Tissue Wet Weight (g)/Body Weight (kg). The brain organ index was calculated for each experimental group of rats.

### 2.8 RNA sequencing (RNA‐Seq)

**FIGURE 3:**
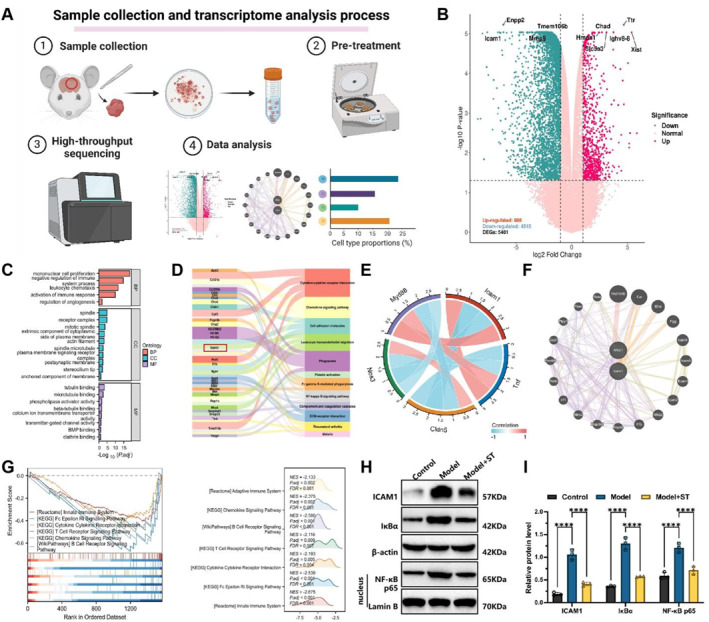
Transcriptomic analysis of rat internal carotid artery severe stenosis model before and after stent implantation. (A) Experimental flowchart illustrating the collection of brain tissue samples and transcriptomic analysis process before and after stent implantation in rats; (B) differential gene expression analysis results, showing the gene expression differences between brain tissue samples before and after stent implantation; (C, D) clusterProfiler enrichment analysis results, displaying the enrichment of DEGs in gene ontology and Kyoto encyclopedia of genes and genomes pathways; (E) correlation analysis between ICAM1 and genes related to inflammation, BBB function, and cerebral microcirculation; (F) protein–protein interaction network analysis results, demonstrating the central role of ICAM1 in the network and its interactions with other inflammation‐related proteins; (G) gene set enrichment analysis enrichment analysis results, indicating significant enrichment of the NF‐κB signaling pathway in DEGs after stent implantation; (H, I) western blot results, showing the nuclear translocation of NF‐κB p65 and changes in the expression levels of ICAM1 and IκBα after stent implantation. All data are presented as mean ± standard error. Experiments were repeated three times, and statistical analysis was performed using ANOVA with Tukey post hoc test. *p < 0.05, **p < 0.01, ***p < 0.001, ****p < 0.0001. DEGs, differentially expressed genes.

The transcriptome sequencing workflow is summarized in Figure 3A. To comprehensively analyze gene expression profiles in rat brain tissue, RNA‐Seq was performed on brain samples from three rats in the model group (ligation only) and three rats in the control group. Total RNA extraction was accomplished with the help of Trizol reagent (15596026, Invitrogen, USA), followed by concentration and purity assessment using a Nanodrop 2000 spectrophotometer (1011U, Thermo Fisher Scientific, USA). RNA samples were considered suitable for library construction if they exhibited an RNA integrity number (RIN) ≥ 7.0 and a 28S:18S ratio ≥1.5. Qualified samples were subjected to library preparation and sequenced on an Illumina HiSeq 2500 system (Illumina, USA) at a depth of 30 million reads per sample. After sequencing, the raw data were quality‐controlled employing FastQC software (v0.11.9) to ensure the quality of paired‐end reads. To remove technical noise, we employed Trimmomatic software (v0.39) to trim Illumina adapters and poly(A) tails. Next, BBMap software (v38.90) was used to correct paired‐end sequences and ensure data integrity. Finally, the high‐quality cleaned reads were aligned to the rat genome reference (GRCm38) utilizing HISAT2 software (v2.2.1), ensuring data accuracy and alignment efficiency.

### 2.9 Differential gene expression analysis

Following normalization of the gene expression data, differential expression analysis was implemented using the “DESeq2” package (v1.42.1) implemented in R. To minimize false‐positive results, the false discovery rate (FDR) correction method was applied to adjust the *p*‐values. Genes with an FDR <0.05 and |logFC| > 1 were considered significantly differentially expressed. Subsequently, heatmaps were generated using the “pheatmap” package (v1.0.12), and volcano plots were created using the “ggplot2” package (v3.3.5) to visualize the expression patterns of the DEGs.

### 2.10 Gene ontology (GO) and Kyoto encyclopedia of genes and genomes (KEGG) enrichment analysis

Functional enrichment of DEGs was assessed by GO and KEGG analyses using the *ClusterProfiler* package in R. GO terms were annotated through the enrichGO function and KEGG pathways using the enrichKEGG function. Enrichment results were filtered utilizing a significance cutoff of *p* < 0.05 to highlight the most relevant functional categories and signaling pathways. This analysis also assessed the enrichment of DEGs in pathways related to inflammatory response, BBB function, and cerebral microcirculation regulation.

### 2.11 Protein‐protein interaction (PPI) network construction

PPI network analysis of DEGs was implemented utilizing the STRING database (https://string‐db.org/). Leveraging the STRINGdb resource through R, a protein interaction landscape was reconstructed based on the DEGs, within which ICAM1 emerged as a functionally relevant node, warranting further scrutiny. Additionally, interactions between ICAM1 and other inflammation‐related proteins, such as vascular cell adhesion molecule 1 (VCAM1), selectin E (SELE), and platelet endothelial cell adhesion molecule 1 (PECAM1), were analyzed to explore their potential role in BBB function and inflammatory responses.

### 2.12 Western Blot

To assess the protein expression levels of ICAM1, nuclear factor kappa‐B (NF‐κB) p65, IκBα, β‐actin, Lamin B, occludin, and ZO‐1, a western blot analysis was performed. Lysis of cellular and brain tissue samples was carried out using RIPA buffer (P0013C, Beyotime, China) enriched with a protease inhibitor cocktail (11836170001, Roche, Switzerland), with incubation maintained for 30 min to ensure adequate protein extraction. The lysates were then centrifuged at 12,000 rpm for 10 min, and the supernatant was collected for protein concentration determination using the BCA assay (23227, Thermo Fisher Scientific, USA). Proteins (20 μg per lane) were fractionated by SDS‐PAGE, electroblotted onto PVDF membranes (Millipore, USA), and subjected to a 1‐h blocking step using 5% non‐fat milk. The following primary antibodies were applied: ICAM1 (1:1000, ab282575, Abcam, UK), NF‐κB p65 (1:1000, 8242S, Cell Signaling Technology [CST], USA), IκBα (1:1000, 4814S, CST, USA), β‐actin (1:5000, ab8226, Abcam, UK), Lamin B (1:1000, 13435S, CST, USA, for nuclear protein detection), occludin (1:1000, ab224526, Abcam, UK), and ZO‐1 (1:1000, ab221547, Abcam, UK). Incubation was carried out overnight at 4°C. To analyze NF‐κB p65 and Lamin B, which are detected in nuclear proteins, nuclear and cytoplasmic fractions were separated utilizing a nuclear protein extraction kit (P0027, Beyotime, China). Nuclear protein extracts were then subjected to SDS‐PAGE and membrane transfer for immunoblotting following established protocols. HRP‐labeled secondary antibodies (1:5000, BA1050, Boster, China) were incubated with the membranes at 37°C for 1 h following primary antibody exposure. Chemiluminescent signals were developed utilizing ECL reagents (Thermo Fisher Scientific, 32209), and band intensities were captured using a Bio‐Rad ChemiDoc MP system.

### 2.13 CBF measurement

To measure CBF, rats were anesthetized with 5% isoflurane, and the skull was thinned surgically until the cortical vasculature was clearly visible. CBF was then measured using the PeriCam PSI laser speckle imaging system and analyzed with PIM software. CBF was recorded preoperatively, as well as on days 21 and 28 post‐surgery. During measurements, rats remained under anesthesia with the cranial window kept intact. The entire cortical surface was scanned, and data were processed using PIM software to visualize blood perfusion changes.

### 2.14 ELISA

To detect the expression levels of tumor necrosis factor‐alpha (TNF‐α), interleukin‐6 (IL‐6), MMP‐9, and ICAM‐1 in brain tissues, samples from each group were collected and rinsed with ice‐cold phosphate‐buffered saline (PBS) prior to homogenization. Tissue homogenates were prepared in RIPA lysis buffer (Beyotime, China) enriched with protease inhibitors (Roche, Switzerland), followed by centrifugation at 12,000 rpm for 10 min. Supernatants were harvested, and protein concentrations were examined utilizing the BCA method (Thermo Fisher). ELISA assays for TNF‐α (ab236712, Abcam, UK), IL‐6 (ab234570, Abcam, UK), MMP‐9 (R6000B, R&D Systems, USA), and ICAM‐1 (RIC100, R&D Systems, USA) were implemented as per the manufacturers' protocols. Protein input ranged from 20 to 50 μg per well, and absorbance was tested utilizing a BioTek microplate reader. Final concentrations were calculated using standard curves.

### 2.15 Cell culture and treatment

**FIGURE 4:**
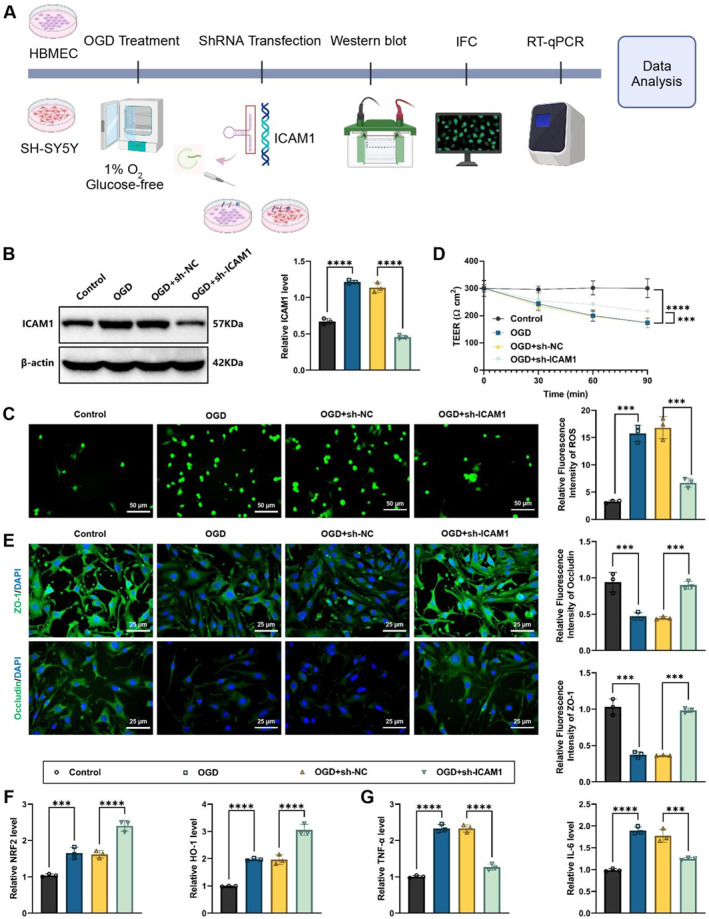
Protective effects of ICAM1 knockdown on HBMEC and SH‐SY5Y cells under OGD conditions. (A) Schematic diagram of the experimental procedure, showing the experimental design for ICAM1 knockdown and OGD treatment; (B) western blot analysis of ICAM1 expression in HBMECs; (C) detection of reactive oxygen species levels in HBMECs using DCF‐DA fluorescence probe; (D) time‐dependent changes in transendothelial electrical resistance (Ω▪cm^2^) values of HBMECs over 90 min; (E) immunofluorescence staining of tight junction proteins (ZO‐1 and occludin) in HBMECs; (F) RT‐qPCR analysis of antioxidant‐related genes (Nrf2 and HO‐1) expression in HBMECs; (G) RT‐qPCR analysis of inflammatory cytokines (TNF‐α and IL‐6) expression in HBMECs. All data are presented as mean ± standard error of the mean. Experiments were repeated three times, and statistical analysis was performed using ANOVA followed by Tukey's post‐hoc test. *p < 0.05, **p < 0.01, ***p < 0.001, ****p < 0.0001. HBMEC, human brain microvascular endothelial cell; OGD, oxygen‐glucose deprivation; RT‐qPCR, reverse transcription quantitative polymerase chain reaction.

Human brain microvascular endothelial cells (HBMECs, Cell System, ACBRI376, USA) and human neuroblastoma cells (SH‐SY5Y cell line, ATCC, CRL‐2266, USA) were selected for this study, which were cultured in high‐glucose DMEM (11995‐065, Gibco, USA) enriched with 10% fetal bovine serum (FBS) (10099141, Gibco, USA) and 1% penicillin–streptomycin (15140122, Gibco, USA) under conditions of 37°C and 5% CO_2_. To simulate severe ICAS, cells were exposed to oxygen–glucose deprivation (OGD) treatment. This involved culturing the cells in glucose‐free DMEM in a hypoxic incubator (1% O_2_, 94% N_2_, 5% CO_2_, Thermo Fisher Scientific, USA) for 6 h.^24^ The in vitro cell experiment design is illustrated in Figure 4A.

### 2.16 Short hairpin RNA (shRNA)‐mediated ICAM1 knockdown

The shRNA technique was employed to knock down the ICAM1 gene. Specifically, a shRNA sequence targeting ICAM1 was designed and cloned into the pLKO.1 vector (psPAX2, Addgene, USA). Viral packaging was carried out using a lentiviral packaging system, and the shRNA plasmids were transfected into HBMEC and SH‐SY5Y cells utilizing Lipofectamine 2000 (11668027, Invitrogen, USA). After 48 h post‐transfection, positive cells were selected by puromycin (purmoycin selection). The resulting cell lines were grouped into the sh‐NC (empty vector transfection) and sh‐ICAM1 (transfected with sh‐ICAM1 lentivirus) groups. To confirm the knockdown efficiency of shRNA, reverse transcription quantitative polymerase chain reaction (RT‐qPCR) and western Blot analyses were implemented to validate the reduction in ICAM1 expression.

### 2.17 Reactive oxygen species (ROS) detection

The levels of ROS in cells were examined utilizing the 2′,7′‐dichlorodihydrofluorescein diacetate (DCF‐DA) fluorescence probe. Cells were cultured to the logarithmic growth phase following standard procedures. The culture medium was deprived of FBS and then incubated with DCF‐DA solution (10 μM, D6883, Sigma‐Aldrich, USA) for 30 min at 37°C under 5% CO_2_. DCF‐DA is esterified inside the cells, where it is converted into the green fluorescent DCF in an ROS‐enriched microenvironment. After incubation, cells were subjected to PBS washes thrice to remove any unbound probe. Fluorescence intensity was then measured using either flow cytometry (FACS) or fluorescence microscopy (Zeiss, Germany). For FACS, fluorescence channels were set to detect the green fluorescence of DCF, with excitation at 488 nm and emission at 525 nm. Changes in ROS levels were reflected by variations in fluorescence intensity.

### 2.18 Measurement of transendothelial electrical resistance (TEER) in HBMECs

TEER was tested utilizing an epithelial voltage–ohm meter to assess the integrity and tightness of HBMEC monolayers. HBMEC or SH‐SY5Y cells (4.5 × 10^4^ cells/cm^2^ ^25^) were seeded on Transwell inserts with polycarbonate membranes. The upper chamber received 600 μL and the lower chamber 1 mL of DMEM. TEER was measured using the Millicell® ERS‐2 electrode (Merck, USA), placed vertically in both chambers and immersed in the medium. The baseline resistance (TEER_blank_) was first recorded. TEER was then measured at 30, 60, and 90 min (TEER_c_) post‐treatment to assess changes in barrier function.

The cells were grouped into control group: HBMEC or SH‐SY5Y cells cultured under normal conditions; OGD group: exposed to OGD; OGD + sh‐NC group: transfected with control shRNA (sh‐NC) and subjected to OGD; OGD + sh‐ICAM1 group: transfected with ICAM1‐targeting shRNA (sh‐ICAM1) and subjected to OGD.

TEER values (Ω·cm^2^) were derived using the following equation: TEER = (TEERc − TEER_blank_) × S(selective membrane area), where S represents the area of the selective membrane. Each experiment was performed in at least two replicates, and results are reported as the mean ± standard error of the mean (SEM).

### 2.19 Immunofluorescence (IF) staining

To evaluate the distribution of ZO‐1 and occludin in HBMECs, cells were cultured on sterile glass coverslips placed in 6‐well plates until reaching 70%–80% confluency. Cells were then fixed with 4% paraformaldehyde (Sigma‐Aldrich, USA) for 10–15 min, followed by PBS washes. Permeabilization of the plasma membrane was performed using 0.1% Triton X‐100 (Sigma‐Aldrich, USA) for 5–10 min. To suppress nonspecific immunoreactivity, cells were blocked with 1% bovine serum albumin (BSA; Sigma‐Aldrich, USA) for 1 h at ambient temperature. Primary antibodies against ZO‐1 (1:100, ab221547, Abcam, UK) and occludin (1:200, ab224526, Abcam, UK) were applied and incubated overnight at 4°C. The next day, cells were rinsed and incubated in the dark for 2 h with Alexa Fluor® 488‐conjugated goat anti‐rabbit IgG (1:100, ab150077, Abcam, UK) as the secondary antibody. Nuclear counterstaining was carried out using DAPI (1:200, Sigma‐Aldrich, USA) for 10 min. Finally, coverslips were mounted with an antifade medium (Vectashield, USA). Fluorescence signals were observed using a Zeiss microscope (Zeiss, Germany), with ZO‐1 and occludin fluorescence appearing green and DAPI‐stained nuclei appearing blue.

### 2.20 RT‐qPCR

Total RNA was isolated from HBMECs and SH‐SY5Y cells using TRIzol reagent (Invitrogen, USA), and sample integrity was preliminarily verified using spectrophotometry, with OD260/280 ratios consistently falling within the accepted 1.8–2.0 range. Reverse transcription into cDNA was initiated using the PrimeScript^TM^ RT Reagent Kit (Takara, Japan), employing a 15‐min incubation at 37°C to facilitate efficient template synthesis, followed by a brief 85°C step to terminate the reaction. Subsequent quantification of transcript levels was achieved using SYBR® Green‐based qPCR (SYBR® Premix Ex Taq^TM^ II, Takara, Japan), carried out on the ABI 7500 platform (Thermo Fisher Scientific, USA). To ensure technical consistency, each reaction was executed in three independent runs. Expression data were normalized to β‐actin and analyzed using the 2^–ΔΔCt^ approach to determine relative fold changes. Primer information is provided in Supporting Information S1: Table S1.

### 2.21 Cell proliferation assay

**FIGURE 5:**
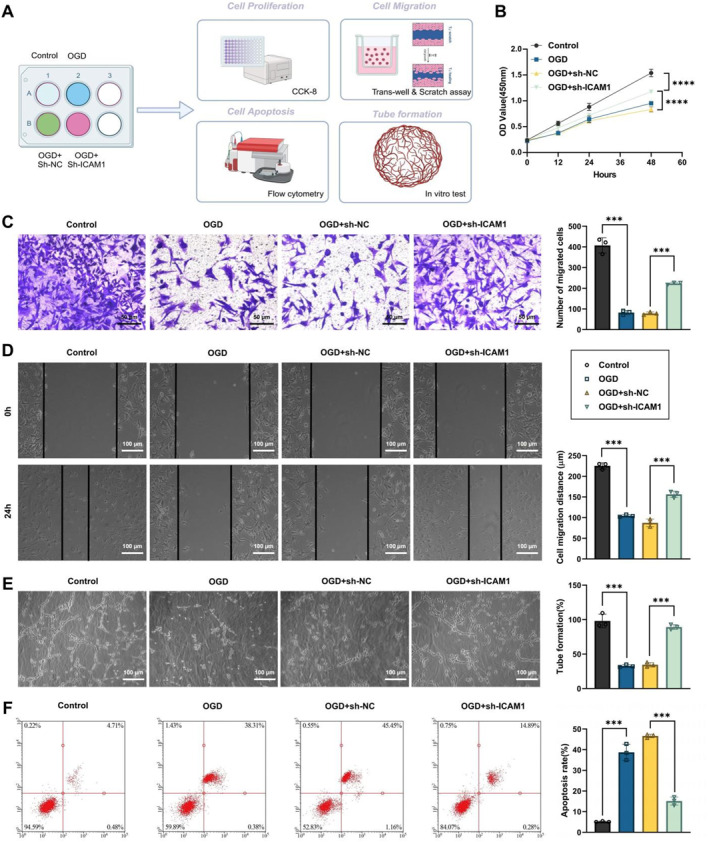
Effect of ICAM1 knockdown on HBMEC function. (A) Schematic diagram of the experimental procedure, illustrating the experimental design for ICAM1 knockdown and oxygen–glucose deprivation treatment in HBMEC and SH‐SY5Y cells; (B) CCK‐8 assay to assess HBMEC cell proliferation; (C) Transwell migration assay to evaluate HBMEC cell migration capacity; (D) scratch assay to measure the migration rate of HBMEC cells; (E) tube formation assay to assess the in vitro angiogenic ability of HBMECs; (F) annexin V‐FITC/PI flow cytometry to determine the apoptosis rate of HBMEC cells. Data are presented as mean ± standard error of the mean. All experiments were performed in triplicate, and statistical analysis was conducted using ANOVA followed by Tukey's post hoc test. *p < 0.05, **p < 0.01, ***p < 0.001, ****p < 0.0001. HBMEC, human brain microvascular endothelial cell.

To evaluate how ICAM1 silencing influences the proliferative capacity of HBMEC and SH‐SY5Y cells, cell viability was monitored using the Cell Counting Kit‐8 (CCK‐8) assay (CK04, Dojindo, Japan). Cells were plated in 96‐well plates at a density of 2 × 10^3^ per well. Following OGD exposure, 10 μL of CCK‐8 solution was introduced into each well, and the plates were incubated at 37°C for 2 h. Absorbance at 450 nm was subsequently recorded employing a microplate reader (Thermo Fisher Scientific, USA), and proliferation rates were inferred based on optical density measurements. The workflow and sample grouping for this analysis are illustrated in Figure 5A.

### 2.22 Transwell migration assay

To further investigate the effect of ICAM1 knockdown on cell migration, a Transwell migration assay was performed. Briefly, bEnd.3 cells were introduced into the upper chamber of a Transwell insert (Corning, USA) (5 × 10^4^ cells per well), 48 h post‐transfection. The lower chamber was supplemented with 600 μL of DMEM containing 10% FBS (Gibco, USA) to serve as a chemoattractant. The assembled system was maintained under standard culture conditions (37°C, 5% CO_2_) for 24 h to allow migration. Following incubation, cells remaining on the upper surface of the membrane were carefully removed with a sterile cotton swab. Migrated cells adhering to the underside were fixed with 4% paraformaldehyde (Sigma‐Aldrich, USA) for 10–15 min, stained using 0.1% crystal violet (Sigma‐Aldrich, USA) for 15 min, and rinsed thoroughly with PBS to eliminate unbound dye. Quantification of migrated cells was conducted microscopically (Zeiss, Germany). Each condition was tested in a minimum of three independent experiments, with results reported as mean ± standard deviation. Statistical comparisons were implemented usng the Student's *t*‐test or one‐way ANOVA where appropriate.

### 2.23 FACS

Annexin V‐FITC/PI–based flow cytometric analysis was utilized to map apoptotic profiles in HBMEC and SH‐SY5Y cells following ICAM1 knockdown. After 48 h of transfection, bEnd.3 cells were trypsinized with 0.25% trypsin (Gibco, USA), rinsed with PBS, and resuspended in 500 μL of Annexin V binding buffer (BD Biosciences, USA). Subsequently, 5 μL each of FITC‐conjugated Annexin V and PI solution (BD Biosciences, USA) was supplemented to the suspension, followed by a 15‐min incubation in the dark at ambient temperature. Samples were immediately subjected to flow cytometric acquisition using the BD FACSCanto II system (USA), and data were processed with FlowJo software. The proportions of early apoptotic cells (Annexin V^+^/PI^−^) and late apoptotic cells (Annexin V^+^/PI^+^) were quantified. Each condition was tested in at least three independent replicates, and results are summarized as mean ± standard deviation. Statistical comparisons were implemented using the Student's *t*‐test or one‐way ANOVA, as appropriate.

### 2.24 Capillary tube formation assay

To assess tube formation ability, HBMEC cells were harvested and seeded into a 96‐well plate pre‐coated with Matrigel (BD Biosciences) (5 × 10^3^ cells per well). The cells were incubated under normal culture conditions. After treatment, the cells were incubated for 24 h and then further cultured for 4 h. Cell morphology was observed employing a phase contrast inverted microscope (100X magnification). The formation of tubular structures was photographed for analysis. For quantitative analysis of tube length, ImageJ software combined with the Angiogenesis Analyzer plugin was used for measurement and analysis.

### 2.25 Cell scratch assay

To evaluate cell migration in vitro, we performed Transwell and wound‐healing assays as shown in Figure 5A. HBMEC cells were initially seeded in 6‐well plates to achieve 70%–80% confluence. A sterile pipette tip or cell scraper was then used to create a straight‐line scratch across the monolayer. The cells within the scratched area were removed, and the well was rinsed once with PBS to eliminate any remaining cells. Fresh medium was added to continue the culture, along with the relevant treatment according to the experimental design. The cells were incubated at 37°C in a 5% CO_2_ incubator, and images of the scratched area were taken at 0 and 24 h using a microscope (Zeiss, Germany) to observe cell migration. Scratch width was quantified to evaluate migratory behavior, with measurements gathered from at least three independent experiments. Results are reported as mean ± standard deviation.

### 2.26 Statistical analysis

Data are summarized as mean ± standard deviation. Differences between control and experimental groups were analyzed using an independent samples *t*‐test (Student's *t*‐test). For comparisons involving multiple groups, one‐way ANOVA was conducted, followed by Tukey's post hoc test for pairwise comparisons. For repeated measures data, two‐way ANOVA was used to assess the effects of treatment and time on the outcomes. In cases where data deviated from a normal distribution, nonparametric alternatives—including the Mann–Whitney *U* test and Kruskal–Wallis test—were used. Statistical analyses were implemented utilizing GraphPad Prism 9.0 (GraphPad Software, USA) or SPSS 22.0 (IBM, USA). A threshold of *p* < 0.05 was adopted to define statistical significance.

## 3 RESULTS

### 3.1 Stent implantation effectively improves vascular dysfunction and hemodynamic abnormalities induced by severe ICAS

In this study, a unilateral severe ICAS model was developed in SD rats, confirmed by ultrasound to exhibit >80% arterial narrowing and associated neurological dysfunction. Early stent implantation (defined as stenting within 7 days of ICAS modeling, in contrast to delayed interventions typically conducted after 2–4 weeks) was performed to evaluate its effects on hemodynamics, pathology, cognitive function, and brain volume (Figure 2A). H&E staining revealed that in the stent implantation group, the arterial lumen significantly expanded, and the thickness of the vascular wall in the stenotic region was markedly reduced, indicating that stent implantation effectively improved the structural vascular lesions caused by severe ICAS (Figure 2B). Doppler ultrasonography showed that after stent implantation, the PSV of the ICA in rats evidently increased, and the RI of blood flow notably decreased, highlighting that the stent improved vascular patency and restored hemodynamic function (Figure 2C,D).

In functional assessments, the MWM test demonstrated that rats in the stent implantation group had a distinctly shorter mean escape latency when searching for the platform, indicating better learning and memory abilities, which suggests that stent implantation effectively alleviated cognitive dysfunction caused by hypoperfusion (Figure 2E). The balance beam test showed that rats in the stent implantation group exhibited significantly better motor coordination than those in the model group without stent implantation, indicating that stent placement contributed to the recovery of motor function (Figure 2F). Furthermore, post‐mortem brain volume index assessments showed a notable decline in brain volume index in the model group, reflecting brain atrophy caused by severe ICAS. In contrast, the brain volume index in the stent implantation group was remarkably higher than that in the model group, suggesting that stent implantation partially improved CBF and alleviated brain tissue atrophy, although it did not fully restore the brain volume to normal levels (Figure 2G).

In conclusion, early stent implantation effectively improves hemodynamic abnormalities and pathological changes induced by severe ICAS, reduces cerebral ischemic damage, and significantly enhances cognitive function, motor coordination, and brain tissue protection in rats.

### 3.2 RNA‐Seq reveals the molecular mechanism by which stent placement improves severe ICAS and reduces the risk of cerebral infarction through regulation of ICAM1

We conducted transcriptomic analysis on brain tissue samples collected from a rat model of severe ICAS before and after stent placement, as well as from healthy rats without stenosis (Figure 3A). Transcriptomic profiling identified marked alterations in gene expression between brain tissues collected pre‐ and post‐stent. The volcano plot (Figure 3B) identified 5401 DEGs, including 886 upregulated genes and 4515 downregulated genes. Notable genes such as ICAM1, Hmox1, Mmp9, Ttr, and Slc5a3 were identified as playing key roles in cerebrovascular and neural repair processes following stent placement.

GO and KEGG enrichment analyses discovered that these DEGs were mainly enriched in pathways related to inflammatory response, BBB function, and cerebral microcirculation regulation (Figure 3C,D). Notably, ICAM1 was significantly enriched in phagosome, NF‐κB signaling, cell adhesion molecules, and leukocyte transendothelial migration pathways, highlighting its key role in regulating local inflammatory responses following stent implantation. Correlation analysis further showed that ICAM1 was strongly positively correlated with inflammation‐related genes such as Myd88 and Tnf (Figure 3E), whereas it was negatively correlated with genes associated with BBB integrity and microcirculation, including Cldn5 and nitric oxide synthase 3 (Nos3).

PPI network analysis further supports the role of ICAM1 as a key node in regulating inflammation and BBB function. We performed PPI network analysis on DEGs and identified ICAM1's central role in the network (Figure 3F). In the PPI network, ICAM1 functions as a hub between various inflammation‐related proteins, such as IL1B, CXCL1, PDE4B, IRF1, NFKB1, and RELA, which are crucial for BBB function and inflammatory responses.

Gene set enrichment analysis (GSEA) (Figure 3G) revealed that NF‐κB acted as a key downstream effector molecule within the innate immune system, mediating both pathogen detection and the induction of pro‐inflammatory cytokines. The cytokine–cytokine receptor interaction, T cell receptor (TCR), and B cell receptor (BCR) signaling pathways were also enriched, indicating that ICAM1 may interact with these pathways to amplify NF‐κB activation. To further validate the transcriptomic findings, we examined key proteins in the ICAM1–NF‐κB signaling axis by western blot. The results demonstrated that ICAM1 expression was diminished following stent implantation, accompanied by reduced nuclear translocation of NF‐κB p65 and decreased IκBα expression (Figure 3H,I). These findings aligned well with the transcriptomic profiles.

In conclusion, this study, through transcriptomic and bioinformatics analysis, unveils the molecular mechanisms underlying early stent implantation in improving neurovascular unit damage and CMD in severe ICAS, particularly highlighting the significant changes in ICAM1 expression and its role in the NF‐κB pathway as critical factors in reducing the risk of watershed infarcts.

### 3.3 ICAM1 knockdown enhances BBB integrity and reduces inflammatory responses

In this study, OGD treatment in HBMEC and neuroblastoma cells (SH‐SY5Y) simulated the ischemic‐hypoxic conditions induced by severe ICAS. To study the potential of ICAM1 in this process, ICAM1 was knocked down using shRNA technology. Negative control, untreated, and intervention groups at different time points were also included (Figure 4A).

Western blot analysis revealed that ICAM1 knockdown brought about a notable decline in protein expression, confirming the effectiveness of the gene knockdown (Figure 4B and Supporting Information S1: Figure S1A). The level of ROS was assessed using the DCF‐DA fluorescence probe, showing a marked reduction in ROS content in the ICAM1 knockdown group. This suggests that ICAM1 knockdown alleviates oxidative stress and improves the cellular metabolic environment (Figure 4C).

TEER measurements demonstrated a significant decrease in TEER in HBMECs under OGD treatment. However, ICAM1 knockdown effectively mitigated this decline, maintaining the electrical resistance of the endothelial cell monolayer (Figure 4D). This further indicates that ICAM1 knockdown helps protect the integrity and function of the BBB. Additionally, IF staining validated that knockdown of ICAM1 led to a notable elevation in ZO‐1 and occludin in HBMECs, further supporting the role of ICAM1 knockdown in enhancing BBB stability (Figure 4E). Moreover, RT‐qPCR analysis confirmed that ICAM1 knockdown distinctly upregulated the expression of antioxidant‐related genes (Nrf2 and HO‐1) in both HBMECs and SH‐SY5Y cells while downregulating the expression of TNF‐α and IL‐6 (Figure 4F,G and Supporting Information S1: Figure S1B,C).

In summary, ICAM1 knockdown alleviates oxidative stress, suppresses inflammatory cytokine expression, and enhances tight junction protein expression under simulated ischemic‐hypoxic conditions. These effects help maintain the barrier function and integrity of the HBMEC monolayer, ultimately improving BBB function and alleviating CMDs.

### 3.4 ICAM1 knockdown enhances cell proliferation, migration, apoptosis, and BBB function

To further investigate the impact of ICAM1 knockdown on the functionality of HBMECs and neurocytes (SH‐SY5Y cell line), we performed a series of experiments, including assessments of cell proliferation, migration, apoptosis, and tube formation ability (Figure 5A).

CCK‐8 assay demonstrated that ICAM1 knockdown significantly enhanced the proliferation rate of HBMECs and SH‐SY5Y cells under OGD conditions. The proliferation rate in the knockdown group was markedly higher than that in the control group (Figure 5B and Supporting Information S1: Figure S1D). This suggests that ICAM1 knockdown offers protective effects on cell survival and proliferation in hypoxic and glucose‐deprived environments.

To assess cell migration, we conducted both Transwell migration and wound healing assays. Transwell assay uncovered that ICAM1 knockdown facilitated the migratory capacity of HBMECs, as evidenced by a substantial increase in the number of migrating cells (Figure 5C), which was validated by the wound healing assay, where ICAM1‐knockdown HBMECs significantly closed the wound area within 24 h, with a higher migration rate relative to the control group (Figure 5D). Collectively, ICAM1 knockdown promotes the migratory function of HBMECs, potentially aiding in the repair and regeneration of endothelial cells.

The angiogenic capacity of HBMECs was assessed through tube formation assays, which demonstrated that knockdown of ICAM1 evidently enhanced the formation of tubular structures by HBMECs in vitro, with a marked increase in both the number of branches and the tubular network, further confirming the positive role of ICAM1 knockdown in promoting endothelial cell angiogenesis under hypoxic conditions (Figure 5E).

Cell apoptosis assessment verified that ICAM1 knockdown remarkably reduced the apoptosis rate of both OGD‐treated HBMECs and SH‐SY5Y cells. Compared to the control group, the knockdown group exhibited a substantial decrease in apoptosis (Figure 5F and Supporting Information S1: Figure S1E).

In summary, ICAM1 knockdown, under hypoxic and glucose‐deprived conditions simulating severe stenosis of the ICA, significantly improved the function of brain microvascular endothelial cells and neural cells by promoting cell proliferation and migration, enhancing angiogenic capacity, and reducing apoptosis. These in vitro results further support ICAM1 as an important molecular target for improving CMD and alleviating neurovascular unit damage.

### 3.5 In vivo Validation of Early Stent Placement in Improving Cerebral Microcirculation, Cognitive Function, and BBB Integrity in Rats with Severe ICAS via ICAM1 Regulation

**FIGURE 6:**
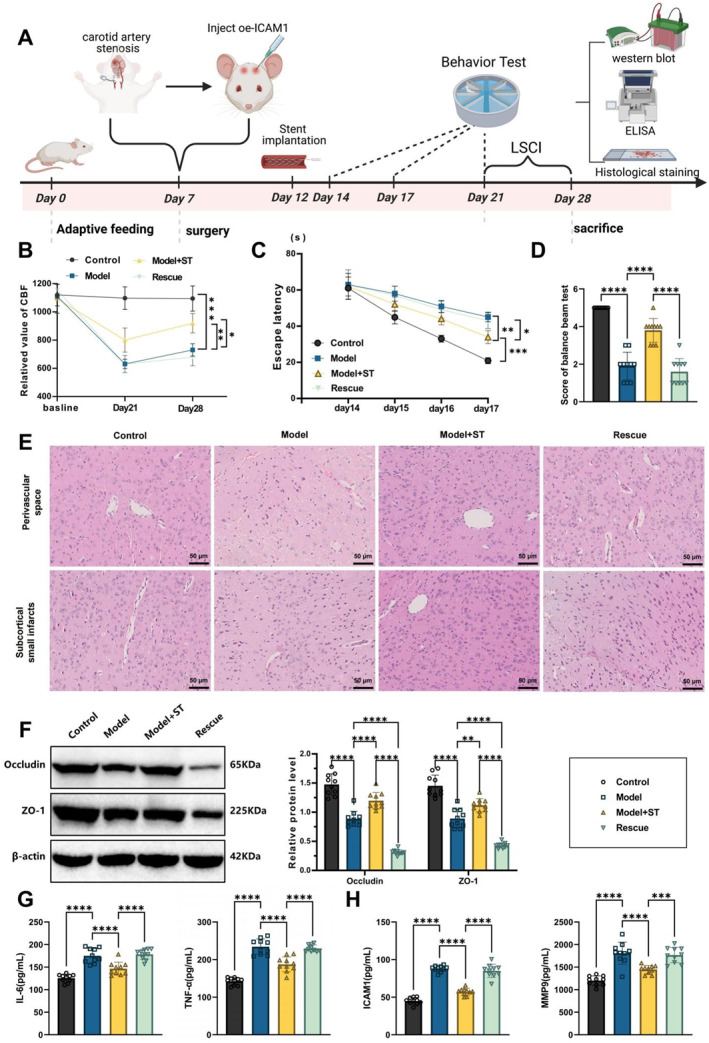
Effects of stent implantation on cerebral microcirculation, cognitive function, and BBB in rats with severe internal carotid artery stenosis. (A) Diagram illustrating experimental groups and protocol; (B) laser speckle contrast imaging measurement of cerebral blood flow in rats; (C) Morris water maze test to assess spatial learning and memory in rats; (D) balance beam test to evaluate motor coordination in rats; (E) H&E staining to observe pathological changes in rat brain tissue structure; (F) western blot analysis of the expression levels of BBB tight junction proteins occludin and ZO‐1 in rat brain tissue; (G, H) ELISA measurement of inflammatory cytokines (TNF‐α, IL‐6) and the expression levels of ICAM1 and MMP‐9 in rat brain tissue. Data are presented as mean ± standard error of the mean, with experiments repeated three times and 10 rats per group. Statistical analysis was performed using ANOVA followed by Tukey's post‐hoc test. *p < 0.05, **p < 0.01, ***p < 0.001, ****p < 0.0001.

The rats were randomized into control, model, stent placement, and rescue groups. The control group consisted of healthy rats with no intervention; the model group underwent surgical induction of an ICAS model; the stent placement group received stent placement therapy in addition to the model; and the rescue group was treated with overexpression of ICAM1 in conjunction with stent placement to evaluate its effect on treatment outcomes (Figure 6A).

LSCI was used to assess changes in cerebral microcirculation, primarily by quantifying CBF. The results demonstrated a notable elevation in CBF in the stent placement group, indicating effective restoration of CBF, marked improvement in microcirculatory conditions, and near‐normal levels of vascular permeability. In contrast, the CBF value in the rescue group was evidently lower than that in the stent placement group, suggesting reduced CBF and increased vascular permeability. This indicates that ICAM1 overexpression interfered with microcirculatory recovery, hindering improvements in blood perfusion (Figure 6B).

We then focused on spatial learning and memory, as well as motor coordination. The MWM results uncovered that the stent placement group had a significantly shorter escape latency, indicating improved cognitive function. The balance beam test further showed that the stent placement group exhibited enhanced motor coordination. Conversely, rats in the rescue group displayed longer escape latencies and poorer motor coordination, further confirming that ICAM1 overexpression hindered the therapeutic effect of stent placement and impaired the recovery of cognitive function and motor coordination (Figure 6C,D).

The H&E staining results demonstrated that in the scaffold implantation group, the brain tissue structure of the rats was close to normal, with a significant reduction in damage to the neurovascular unit and effective improvement in cerebral microcirculation. In contrast, the rescue group exhibited more pronounced pathological damage in the brain tissue, with exacerbated disruption of the neurovascular unit, suggesting that overexpression of ICAM1 worsened brain tissue injury and ischemia (Figure 6E).

Western blot and immunohistochemistry analyses revealed that in the scaffold implantation group, the expression levels of occludin and ZO‐1 were elevated, indicating that scaffold implantation treatment helped restore the integrity of the BBB and alleviated vascular damage induced by ischemia and hypoxia. In the rescue group, however, the expression levels of occludin and ZO‐1 were distinctly decreased, suggesting that ICAM1 overexpression exacerbated the damage to the BBB and hindered vascular repair (Figure 6F).

Furthermore, ELISA measurements of TNF‐α and IL‐6 in the brain tissue showed a significant reduction in inflammatory factor levels in the scaffold implantation group, whereas the rescue group displayed markedly elevated levels, indicating that ICAM1 overexpression intensified the inflammatory response. Additionally, the expression of ICAM1 and MMP‐9 was evidently upregulated in the rescue group, which strongly correlated with increased BBB disruption and enhanced inflammation (Figure 6G,H).

In conclusion, scaffold implantation treatment promotes cerebral microcirculation repair and functional recovery by improving CBF, restoring BBB integrity, and alleviating inflammatory responses and neurovascular unit damage. In contrast, ICAM1 overexpression, by upregulating MMP‐9 expression, exacerbates BBB damage and inflammation, thus interfering with the therapeutic effects of scaffold implantation.

## 4 DISCUSSION

Our study focused on the molecular mechanisms underlying CMDs and BBB dysfunction induced by ICAS, with a particular focus on the significance of ICAM1. ICAS is a severe condition that significantly impacts cerebral hemodynamics, increasing the risk of watershed infarctions. Previous studies have primarily concentrated on the diagnosis and pharmacological treatment of ICAS.^26^, ^27^, ^28^, ^29^, ^30^ Although stenting, a key interventional therapy, has shown promising effects in restoring blood flow, the specific molecular mechanisms remain incompletely understood. This study, employing transcriptomics, in vitro and in vivo experiments, and multi‐level validation, provides the first systematic elucidation of ICAM1's dual role in early stent therapy, offering a comprehensive analysis of its function in BBB repair and inflammation regulation. These findings contribute novel insights and a theoretical basis for further investigations into the molecular underpinnings of this process.

Our results demonstrate that early stent placement significantly alleviates the cerebral microcirculatory disturbances, BBB damage, and cognitive dysfunction associated with ICAS. In experimental models, stenting notably enhanced CBF, reduced inflammation, and restored BBB integrity. These findings align with previous reports on stenting's effectiveness in restoring cerebral hemodynamics.^31^, ^32^ However, our study further uncovers the profound molecular‐level effects of stent placement on neurovascular unit repair. Additionally, behavioral testing revealed that stent therapy improved cognitive function, providing new evidence for its clinical value and addressing a gap in previous research on its effects on neurocognitive outcomes.

As an inflammation‐associated molecule, ICAM1 has attracted growing attention for its role in BBB repair. By integrating transcriptomic and molecular data, this study demonstrates for the first time the specific mechanism by which ICAM1 regulates BBB function, particularly its impact on the expression of ZO‐1 and Occludin, thereby expanding our understanding of ICAM1 and supporting its potential as a therapeutic target.^33^, ^34^ Further analysis revealed that ICAM1 plays a central role in neurovascular repair during stent implantation by modulating inflammatory pathways, amplifying the NF‐κB signaling cascade, and regulating tight junction proteins. ICAM1 showed significant positive correlations with key inflammatory mediators such as Myd88 and Tnf^35^, ^36^ as well as BBB‐ and inflammation‐related genes, including IL1B, CXCL1, PDE4B, IRF1, NFKB1, and RELA.^37^, ^38^, ^39^, ^40^ These associations suggest that ICAM1 may further activate the NF‐κB pathway through interactions with NFKB1 and RELA, enhancing TNF expression and amplifying the inflammatory response.

Combined PPI network and KEGG pathway analyses suggest that ICAM1 may coordinate with innate and adaptive immune pathways, including cytokine–cytokine receptor, TCR, and BCR signaling pathways, to amplify NF‐κB activation, thereby modulating both immune cell activity and local inflammatory damage. Moreover, ICAM1 expression showed strong negative correlations with Cldn5 and Nos3, genes critical for maintaining BBB integrity and regulating cerebral microcirculation,^41^, ^42^ suggesting that ICAM1 overexpression may exacerbate BBB disruption and increase the risk of watershed infarcts.

Consistently, GSEA results and western blot validation demonstrated that stent implantation downregulated ICAM1 expression and reduced nuclear translocation of NF‐κB p65 and IκBα expression, further supporting its role in CMD regulation. In addition to ICAM1, other hub genes such as Hmox1, Mmp9, Ttr, and Slc5a3 may also contribute to cerebrovascular and neurovascular repair processes post‐stenting.^33^, ^43^, ^44^ Previous studies have documented the crucial role of ICAM1 in various models of neurovascular unit repair, especially those involving inflammatory injury and BBB restoration.^45^, ^46^ These insights collectively highlight the importance of early intervention strategies that target ICAM1 and its downstream NF‐κB signaling to alleviate local inflammation, protect BBB function, and promote brain tissue recovery following ICAS.

Additionally, in vitro experiments further confirmed the critical role of ICAM1 knockdown in improving BBB function. Specifically, ICAM1 knockdown significantly reduced the expression of inflammatory factors while enhancing tight junction protein expression, thereby promoting the proliferation and migration of endothelial and neural cells. These results contrast with previous reports that highlighted the negative role of ICAM1 in inflammation,^47^ emphasizing its protective effect on BBB repair under certain conditions. However, rescue experiments involving ICAM1 overexpression indicated that its excessive activation could impair scaffold therapy outcomes by upregulating MMP‐9 and inflammatory factor expression. This dual‐effect underscores the need for caution in modulating ICAM1 during scaffold therapy to avoid diminishing therapeutic efficacy due to its overactivation.

This study makes another significant contribution by revealing the dual role of ICAM1 in stent therapy and exploring its dynamic regulatory mechanisms under different levels of inflammation. Unlike previous literature that simplistically identifies ICAM1 as a pro‐inflammatory factor, we systematically elucidated its complex role in regulating inflammation, BBB function, and neurovascular unit repair through a series of experiments. This finding not only deepens our understanding of the molecular mechanisms underlying stent therapy but also provides theoretical support for future therapeutic strategies aimed at optimizing treatment by targeting ICAM1.

On a molecular and mechanistic level, this study deepens the understanding of stent implantation therapy, offering theoretical support for the application of precision medicine in severe ICA stenosis. ICAM1, as a potential molecular target, holds promise for optimizing stent implantation outcomes and reducing the incidence of complications such as cerebral infarction. These findings underscore the translational potential of modulating NF‐κB signaling and BBB function in the rational design of novel anti‐inflammatory therapeutics.

Although this study reveals the crucial role of ICAM1 in stent implantation, the experimental model may not fully replicate the complexity of human disease. Additionally, the interactions between ICAM1 and other inflammatory factors or signaling pathways require further investigation. Future research should validate these mechanisms in more complex animal models or clinical trials and explore the potential of combined targeted therapies to further enhance the therapeutic effects of stent implantation, driving innovation and development in cerebrovascular disease interventions. Despite demonstrating the involvement of ICAM1 in the beneficial outcomes of stent implantation, the study is not without limitations that warrant consideration. First, rodent models may not fully replicate the complexity of human cerebrovascular diseases. Notably, there are substantial species‐specific differences in the properties of the BBB and cerebrovascular architecture between humans and mice. For example, the human brain exhibits higher vascular density, more complex branching, and a finer microcirculatory network, whereas the murine vasculature is simpler, potentially affecting drug distribution. Human BBB endothelial cells have tighter junctions (with higher expression of occludin and claudins), greater pericyte coverage (∼90%), and reduced permeability, compared to the more permeable mouse BBB (∼50% pericyte coverage).^48^, ^49^ Furthermore, the human BBB expresses higher levels of nutrient transporters like GLUT1, ensuring selective metabolic uptake, whereas mice display higher expression of efflux transporters such as P‐gp, which may accelerate drug clearance.^50^, ^51^ Additionally, human brain metabolism is more glucose‐dependent, whereas mice demonstrate higher utilization of ketone bodies. Under inflammatory conditions, the mouse BBB tends to become more permeable, whereas human BBB repair involves more complex immune regulatory mechanisms.^52^, ^53^ These physiological and pathological differences indicate that although mouse models are widely used for BBB research, findings must be cautiously validated in clinical settings before translational application, thereby increasing the challenges of clinical translation. Moreover, although clinical data support the long‐term efficacy of stent implantation in improving cerebral perfusion and reducing restenosis risk,^54^ some evidence suggests that stenting may disrupt BBB integrity through hemodynamic alterations.^6^ In this study, although CBF was assessed using Doppler ultrasound and LSCI, we acknowledge the absence of more comprehensive assessments, such as in vivo microscopy or microvascular pressure measurements, due to technical limitations. This may have limited the completeness of our hemodynamic evaluation.

## 5 CONCLUSION

This study systematically elucidates the mechanisms by which early stent implantation improves neurovascular unit injury, CMD, and cognitive dysfunction induced by severe ICA stenosis. Through transcriptomic and bioinformatics analyses, we found that stent implantation significantly downregulates ICAM1 expression, reduces inflammation, restores BBB integrity, and improves CBF, thereby reducing the risk of watershed infarction (watershed infarctions refer to ischemic lesions occurring in border zones between major cerebral arteries, often due to hypoperfusion). Furthermore, both cellular and animal evidence demonstrated that ICAM1 governs BBB integrity, inflammatory mediator release, and microcirculatory repair through the NF‐κB pathway (graphic abstract). Silencing ICAM1 significantly enhances endothelial‐neuronal function in the brain microvasculature, whereas its overexpression impedes the therapeutic effects of stent implantation. This study clarifies the dual role of ICAM1, providing an important molecular basis for the repair of the neurovascular unit after ICA stenosis.

## AUTHOR CONTRIBUTIONS

**Kuo Li:** Conceptualization, methodology, investigation, writing—original draft. **Chuansuo Zhang:** Methodology, validation, data curation. **Li Xuan Wang:** Formal analysis, visualization. **Xiaoxuan Wang:** Investigation, resources. **Ruyue Wang:** Supervision, writing—review and editing. All authors critically reviewed and approved the final manuscript.

## CONFLICT OF INTEREST STATEMENT

The authors declare no conflicts of interest.

## ETHICS STATEMENT

This study strictly adhered to the ethical guidelines and regulations concerning animal experiments and received approval from the Institutional Animal Care and Use Committee (IACUC) of Cangzhou Central Hospital to ensure the rationality and compliance of the experimental protocol (Approval no. 2024‐059‐01).

## CONSENT FOR PUBLICATION

Not applicable.

## Supporting information

Supporting Information S1

## Data Availability

The datasets used or analyzed during the current study are available from the corresponding author on reasonable request.

## References

[1] Chandra, A. , C. R. Stone , W. A. Li , X. Geng , and Y. Ding . 2017. “The Cerebral Circulation and Cerebrovascular Disease II: Pathogenesis of Cerebrovascular Disease.” Brain circulation 3(2): 57–65. 10.4103/bc.bc_11_17. 30276306 PMC6126265

[2] Paulson, O. B 1971. “Cerebral Apoplexy (Stroke): Pathogenesis, Pathophysiology and Therapy as Illustrated by Regional Blood Flow Measurements in the Brain.” Stroke 2(4): 327–360. 10.1161/01.str.2.4.327. 4398838

[3] Hannawi, Y. 2024. “Cerebral Small Vessel Disease: a Review of the Pathophysiological Mechanisms.” Translational stroke research 15(6): 1050–1069. 10.1007/s12975-023-01195-9. 37864643

[4] Weill, C. , L. Suissa , J. Darcourt , and M. H. Mahagne . 2017. “The Pathophysiology of Watershed Infarction: a Three‐Dimensional Time‐of‐Flight Magnetic Resonance Angiography Study.” Journal of Stroke and Cerebrovascular Diseases: The Official Journal of National Stroke Association 26(9): 1966–1973. 10.1016/j.jstrokecerebrovasdis.2017.06.016. 28694111

[5] Szarmach, A. , M. A. Małkiewicz , A. Zdun‐Ryżewska , G. Halena , M. Radkowski , J. Dzierżanowski , K. Chwojnicki , et al. 2019. “Relative Cerebral Blood Transit Time Decline and Neurological Improvement in Patients After Internal Carotid Artery Stenting.” Advances in Experimental Medicine and Biology 1176: 71–80. 10.1007/5584_2019_378. 31098943

[6] Szarmach, A. , G. Halena , M. Kaszubowski , M. Piskunowicz , M. Studniarek , P. Lass , E. Szurowska , and P. J. Winklewski . 2017. “Carotid Artery Stenting and Blood‐Brain Barrier Permeability in Subjects with Chronic Carotid Artery Stenosis.” International Journal of Molecular Sciences 18(5): 1008. 10.3390/ijms18051008. 28481312 PMC5454921

[7] Ogami, R. , T. Nakahara , O. Hamasaki , H. Araki , and K. Kurisu . 2011. “Cerebrospinal Fluid Enhancement on Fluid Attenuated Inversion Recovery Images After Carotid Artery Stenting with Neuroprotective Balloon Occlusions: Hemodynamic Instability and blood‐brain Barrier Disruption.” Cardiovascular and Interventional Radiology 34(5): 936–941. 10.1007/s00270-010-0035-4. 21127870

[8] Mansour, A. , S. Rashad , K. Niizuma , M. Fujimura , and T. Tominaga . 2019. “A Novel Model of Cerebral Hyperperfusion with blood‐brain Barrier Breakdown, White Matter Injury, and Cognitive Dysfunction.” Journal of Neurosurgery 133(5): 1460–1472. 10.3171/2019.7.JNS19212. 31628277

[9] Thompson, P. W. , A. M. Randi , and A. J. Ridley . 2002. “Intercellular Adhesion Molecule (ICAM)‐1, but Not ICAM‐2, Activates RhoA and Stimulates c‐fos and rhoA Transcription in Endothelial Cells.” Journal of immunology (Baltimore, Md: 1950) 169(2): 1007–1013. 10.4049/jimmunol.169.2.1007. 12097408

[10] Qian, W. J. , J. S. Yan , X. Y. Gang , L. Xu , S. Shi , X. Li , F. J. Na , L. T. Cai , H. M. Li , and M. F. Zhao . 2024. “Intercellular Adhesion molecule‐1 (ICAM‐1): from Molecular Functions to Clinical Applications in Cancer Investigation.” Biochimica et biophysica acta. Reviews on cancer 1879(6): 189187. 10.1016/j.bbcan.2024.189187. 39317271

[11] Taftaf, R. , X. Liu , S. Singh , Y. Jia , N. K. Dashzeveg , A. D. Hoffmann , L. El‐Shennawy , et al. 2021. “ICAM1 Initiates CTC Cluster Formation and Trans‐endothelial Migration in Lung Metastasis of Breast Cancer.” Nature Communications 12(1): 4867. 10.1038/s41467-021-25189-z. PMC8358026 34381029

[12] Nordal, R. A. , and C. S. Wong . 2004. “Intercellular Adhesion molecule‐1 and blood‐spinal Cord Barrier Disruption in Central Nervous System Radiation Injury.” Journal of Neuropathology and Experimental Neurology 63(5): 474–483. 10.1093/jnen/63.5.474. 15198126

[13] Frank, P. G. , and M. P. Lisanti . 2008. “ICAM‐1: Role in Inflammation and in the Regulation of Vascular Permeability.” American Journal of Physiology ‐ Heart and Circulatory Physiology 295(3): H926–H927. 10.1152/ajpheart.00779.2008. 18689494 PMC2544488

[14] Dietrich, J. B. 2002. “The Adhesion Molecule ICAM‐1 and Its Regulation in Relation with the blood‐brain Barrier.” Journal of Neuroimmunology 128(1–2): 58–68. 10.1016/s0165-5728(02)00114-5. 12098511

[15] Ding, S. , J. Liu , X. Han , W. Ding , Z. Liu , Y. Zhu , W. Zhan , et al. 2022. “ICAM‐1‐related Noncoding RNA Accelerates Atherosclerosis by Amplifying NF‐κB Signaling.” Journal of Molecular and Cellular Cardiology 170: 75–86. 10.1016/j.yjmcc.2022.06.001. 35714558

[16] Singh, V. , R. Kaur , P. Kumari , C. Pasricha , and R. Singh . 2023. “ICAM‐1 and VCAM‐1: Gatekeepers in Various Inflammatory and Cardiovascular Disorders.” Clinica chimica acta; international journal of clinical chemistry 548: 117487. 10.1016/j.cca.2023.117487. 37442359

[17] Nakashima, Y. , E. W. Raines , A. S. Plump , J. L. Breslow , and R. Ross . 1998. “Upregulation of VCAM‐1 and ICAM‐1 at atherosclerosis‐prone Sites on the Endothelium in the ApoE‐deficient Mouse.” Arteriosclerosis, Thrombosis, and Vascular Biology 18(5): 842–851. 10.1161/01.atv.18.5.842. 9598845

[18] Zhang, R. L. , M. Chopp , N. Jiang , W. X. Tang , J. Prostak , A. M. Manning , and D. C. Anderson . 1995. “Anti‐Intercellular Adhesion molecule‐1 Antibody Reduces Ischemic Cell Damage After Transient but Not Permanent Middle Cerebral Artery Occlusion in the Wistar Rat.” Stroke 26(8): 1438–1443. 10.1161/01.str.26.8.1438. 7631350

[19] Lakhan, S. E. , A. Kirchgessner , and M. Hofer . 2009. “Inflammatory Mechanisms in Ischemic Stroke: Therapeutic Approaches.” Journal of Translational Medicine 7(1): 97. 10.1186/1479-5876-7-97. 19919699 PMC2780998

[20] Robinson, N. B. , K. Krieger , F. M. Khan , W. Huffman , M. Chang , A. Naik , R. Yongle , et al. 2019. “The Current State of Animal Models in Research: a Review.” International Journal of Surgery 72: 9–13. 10.1016/j.ijsu.2019.10.015. 31627013

[21] Regenberg, A. , D. J. Mathews , D. M. Blass , H. Bok , J. T. Coyle , P. Duggan , R. Faden , et al. 2009. “The Role of Animal Models in Evaluating Reasonable Safety and Efficacy for Human Trials of Cell‐based Interventions for Neurologic Conditions.” Journal of Cerebral Blood Flow and Metabolism : Official Journal of the International Society of Cerebral Blood Flow and Metabolism 29(1): 1–9. 10.1038/jcbfm.2008.98. 18728679 PMC2682696

[22] Li, L. , P. R. Krafft , N. Zeng , R. Duan , X. Qi , A. Shao , F. Xue , and J. H. Zhang . 2024. “Microglia Autophagy Mediated by TMEM166 Promotes Ischemic Stroke Secondary to Carotid Artery Stenosis.” Aging and Disease 15(3): 1416–1431. 10.14336/AD.2023.0803. 37611898 PMC11081158

[23] Li, X. , and M. E. Wolf . 2011. “Visualization of virus‐infected Brain Regions Using a GFP‐Illuminating Flashlight Enables Accurate and Rapid Dissection for Biochemical Analysis.” Journal of Neuroscience Methods 201(1): 177–179. 10.1016/j.jneumeth.2011.07.016. 21816173 PMC3176337

[24] Ma, C. , L. Yang , Q. Gao , and L. Wang . 2022. “miR‐602 Activates NRF2 Antioxidant Pathways to Protect HBMECs from OGD/R‐Induced Oxidative Stress via Targeting KEAP1 and HRD1.” Disease Markers 2022: 6967573. 10.1155/2022/6967573. 36193504 PMC9526584

[25] Eigenmann, D. E. , G. Xue , K. S. Kim , A. V. Moses , M. Hamburger , and M. Oufir . 2013. “Comparative Study of Four Immortalized Human Brain Capillary Endothelial Cell Lines, hCMEC/D3, hBMEC, TY10, and BB19, and Optimization of Culture Conditions, for an in Vitro blood‐brain Barrier Model for Drug Permeability Studies.” Fluids and Barriers of the CNS 10(1): 33. 10.1186/2045-8118-10-33. 24262108 PMC4176484

[26] Jogestrand, T. , M. Lindqvist , and J. Nowak . and Swedish Quality Board for Carotid Surgery . 2002. “Diagnostic Performance of Duplex Ultrasonography in the Detection of High Grade Internal Carotid Artery Stenosis.” European Journal of Vascular and Endovascular Surgery : The Official Journal of the European Society for Vascular Surgery 23(6): 510–518. 10.1053/ejvs.2002.1621. 12093067

[27] Kassem, M. , A. Florea , F. M. Mottaghy , R. van Oostenbrugge , and M. E. Kooi . 2020. “Magnetic Resonance Imaging of Carotid Plaques: Current Status and Clinical Perspectives.” Annals of Translational Medicine 8(19): 1266. 10.21037/atm-2020-cass-16. 33178798 PMC7607136

[28] Paraskevas, K. I. , F. J. Veith , H. H. Eckstein , J. B. Ricco , and D. P. Mikhailidis . 2020. “Cholesterol, Carotid Artery Disease and Stroke: what the Vascular Specialist Needs to Know.” Annals of Translational Medicine 8(19): 1265. 10.21037/atm.2020.02.176. 33178797 PMC7607102

[29] Paraskevas, K. I. , G. Hamilton , and D. P. Mikhailidis . 2007. “Statins: an Essential Component in the Management of Carotid Artery Disease.” Journal of Vascular Surgery 46(2): 373–386. 10.1016/j.jvs.2007.03.035. 17664116

[30] Hussain, M. A. , G. Saposnik , S. Raju , K. Salata , M. Mamdani , J. V. Tu , D. L. Bhatt , S. Verma , and M. Al‐Omran . 2018. “Association Between Statin Use and Cardiovascular Events After Carotid Artery Revascularization.” Journal of the American Heart Association 7(16): e009745. 10.1161/JAHA.118.009745. 30369318 PMC6201401

[31] van Laar, P. J. , J. van der Grond , F. L. Moll , W. P. Mali , and J. Hendrikse . 2006. “Hemodynamic Effect of Carotid Stenting and Carotid Endarterectomy.” Journal of Vascular Surgery 44(1): 73–78. 10.1016/j.jvs.2006.03.023. 16730158

[32] Sfyroeras, G. , C. D. Karkos , C. Liasidis , C. Spyridis , A. S. Dimitriadis , K. Kouskouras , and T. S. Gerassimidis . 2006. “The Impact of Carotid Stenting on the Hemodynamic Parameters and Cerebrovascular Reactivity of the Ipsilateral Middle Cerebral Artery.” Journal of Vascular Surgery 44(5): 1016–1022. 10.1016/j.jvs.2006.07.025. 17098536

[33] Bui, T. M. , H. L. Wiesolek , and R. Sumagin . 2020. “ICAM‐1: a Master Regulator of Cellular Responses in Inflammation, Injury Resolution, and Tumorigenesis.” Journal of Leukocyte Biology 108(3): 787–799. 10.1002/JLB.2MR0220-549R. 32182390 PMC7977775

[34] Rakhimbaeva, G. S. , and K. B. K. Abdurakhmonova . 2023. “ICAM‐1 and CRP as Biomarkers of 3‐month Outcome in Acute Ischaemic Stroke.” BMJ Neurology Open 5(2): e000516. 10.1136/bmjno-2023-000516. PMC10749038 38145240

[35] Zhu, G. , Z. Cheng , Y. Huang , W. Zheng , S. Yang , C. Lin , and J. Ye . 2020. “MyD88 Mediates Colorectal Cancer Cell Proliferation, Migration and Invasion via NF‐κB/AP‐1 Signaling Pathway.” International Journal of Molecular Medicine 45(1): 131–140. 10.3892/ijmm.2019.4390. 31746347 PMC6889924

[36] Pasquereau, S. , A. Kumar , and G. Herbein . 2017. “Targeting TNF and TNF Receptor Pathway in HIV‐1 Infection: from Immune Activation to Viral Reservoirs.” Viruses 9(4): 64. 10.3390/v9040064. 28358311 PMC5408670

[37] Alves‐Hanna, F. S. , F. R. P. Silva , D. S. Pereira , A. L. A. B. Leal , F. Magalhães‐Gama , and A. G. Costa . 2024. “Association Between the IL1B‐511 C>T Polymorphism and the Risk of Hematologic Malignancies: Data from a meta‐analysis.” Cancer Biology & Therapy 25(1): 2382503. 10.1080/15384047.2024.2382503. 39039694 PMC11268255

[38] Wang, N. , W. Liu , Y. Zheng , S. Wang , B. Yang , M. Li , J. Song , et al. 2018. “CXCL1 Derived from tumor‐associated Macrophages Promotes Breast Cancer Metastasis via Activating NF‐κB/SOX4 Signaling.” Cell Death & Disease 9(9): 880. 10.1038/s41419-018-0876-3. 30158589 PMC6115425

[39] Cartwright, T. , N. D. Perkins , and C. L Wilson . 2016. “NFKB1: a Suppressor of Inflammation, Ageing and Cancer.” FEBS Journal 283(10): 1812–1822. 10.1111/febs.13627. 26663363

[40] Yang, K. , J. Zhao , S. Liu , and S. Man . 2023. “RELA Promotes the Progression of Oral Squamous Cell Carcinoma via TFAP2A‐Wnt/β‐catenin Signaling.” Molecular Carcinogenesis 62(5): 641–651. 10.1002/mc.23512. 36789977

[41] Yang, Z. , P. Lin , B. Chen , X. Zhang , W. Xiao , S. Wu , C. Huang , D. Feng , W. Zhang , and J. Zhang . 2021. “Autophagy Alleviates hypoxia‐induced blood‐brain Barrier Injury via Regulation of CLDN5 (Claudin 5).” Autophagy 17(10): 3048–3067. 10.1080/15548627.2020.1851897. 33280500 PMC8526012

[42] Man, A. W. C. , Y. Zhou , G. Reifenberg , A. Camp , T. Münzel , A. Daiber , N. Xia , and H. Li . 2023. “Deletion of Adipocyte NOS3 Potentiates high‐fat diet‐induced Hypertension and Vascular Remodelling via Chemerin.” Cardiovascular Research 119(17): 2755–2769. 10.1093/cvr/cvad164. 37897505 PMC10757584

[43] Meng, Z. , H. Liang , J. Zhao , J. Gao , C. Liu , X. Ma , J. Liu , et al. 2021. “HMOX1 Upregulation Promotes Ferroptosis in Diabetic Atherosclerosis.” Life Sciences 284: 119935. 10.1016/j.lfs.2021.119935. 34508760

[44] Zhang, H. , L. Liu , C. Jiang , K. Pan , J. Deng , and C. Wan . 2020. “MMP9 Protects Against LPS‐Induced Inflammation in Osteoblasts.” Innate Immunity 26(4): 259–269. 10.1177/1753425919887236. 31726909 PMC7251795

[45] Otgongerel, D. , H. J. Lee , and S. A. Jo . 2023. “Induction of ICAM1 in Brain Vessels Is Implicated in an Early AD Pathogenesis by Modulating Neprilysin.” NeuroMolecular Medicine 25(2): 193–204. 10.1007/s12017-022-08726-x. 35948857

[46] Yang, C. , B. Ni , L. Shen , Z. Li , L. Zhou , H. Wu , Y. Zhang , et al. 2024. “Systematic pan‐cancer Analysis Insights into ICAM1 as an Immunological and Prognostic Biomarker.” The FASEB Journal: Official Publication of the Federation of American Societies for Experimental Biology 38(13): e23802. 10.1096/fj.202302176R. 38979944

[47] Gross, M. D. , S. J. Bielinski , J. R. Suarez‐Lopez , A. P. Reiner , K. Bailey , B. Thyagarajan , J. J. Carr , D. A. Duprez , and D. R. Jacobs, Jr . 2012. “Circulating Soluble Intercellular Adhesion Molecule 1 and Subclinical Atherosclerosis: the Coronary Artery Risk Development in Young Adults Study.” Clinical Chemistry 58(2): 411–420. 10.1373/clinchem.2011.168559. 22179741 PMC3867124

[48] Hashimoto, Y. , C. Greene , A. Munnich , and M. Campbell . 2023. “The CLDN5 Gene at the blood‐brain Barrier in Health and Disease.” Fluids and Barriers of the CNS 20(1): 22. 10.1186/s12987-023-00424-5. 36978081 PMC10044825

[49] Shen, Q. , Q. Yu , T. Chen , and L. Zhang . 2025. “Rosuvastatin Mitigates blood‐brain Barrier Disruption in sepsis‐associated Encephalopathy by Restoring Occludin Levels.” European Journal of Medical Research 30(1): 103. 10.1186/s40001-025-02314-y. 39953583 PMC11827257

[50] Veys, K. , Z. Fan , M. Ghobrial , A. Bouché , M. García‐Caballero , K. Vriens , N. V. Conchinha , et al. 2020. “Role of the GLUT1 Glucose Transporter in Postnatal CNS Angiogenesis and Blood‐Brain Barrier Integrity.” Circulation Research 127(4): 466–482. 10.1161/CIRCRESAHA.119.316463. 32404031 PMC7386868

[51] Sadiq, M. W. , Y. Uchida , Y. Hoshi , M. Tachikawa , T. Terasaki , and M. Hammarlund‐Udenaes . 2015. “Validation of a P‐Glycoprotein (P‐gp) Humanized Mouse Model by Integrating Selective Absolute Quantification of Human MDR1, Mouse Mdr1a and Mdr1b Protein Expressions with in Vivo Functional Analysis for Blood‐Brain Barrier Transport.” PLoS One 10(5): e0118638. 10.1371/journal.pone.0118638. 25932627 PMC4416786

[52] Kong, G. , W. Xiong , C. Li , C. Xiao , S. Wang , W. Li , X. Chen , et al. 2023. “Treg cells‐derived Exosomes Promote blood‐spinal Cord Barrier Repair and Motor Function Recovery After Spinal Cord Injury by Delivering miR‐2861.” Journal of Nanobiotechnology 21(1): 364. 10.1186/s12951-023-02089-6. 37794487 PMC10552208

[53] Liberale, L. , N. R. Bonetti , Y. M. Puspitasari , A. Vukolic , A. Akhmedov , C. Diaz‐Cañestro , S. Keller , et al. 2021. “TNF‐α Antagonism Rescues the Effect of Ageing on Stroke: Perspectives for Targeting inflamm‐ageing.” European Journal of Clinical Investigation 51(11): e13600. 10.1111/eci.13600. 34076259 PMC8596431

[54] Okmen, E. , and N. Cam . 2002. “Direkt Stent Implantasyonu: Uygulanabilirliği, Avantajlari ve Dezavantajlari [Direct stent implantation: feasibility, advantages and disadvantages].” Anadolu Kardiyoloji Dergisi : AKD = The Anatolian journal of cardiology 2(3): 237–243. 12223333

