# Supplementary material for: Stent treatment improves cerebral microcirculatory disorder and blood–brain barrier function in internal carotid artery stenosis via intercellular adhesion molecule 1 modulation
Source: J Cell Commun Signal. 2025 Dec 14;19(4):e70058. doi: 10.1002/ccs3.70058 (PMC12702815; doi:10.1002/ccs3.70058)
Supplement: Supplementary file 1 — Supporting Information S1 [file CCS3-19-e70058-s001.docx]

**
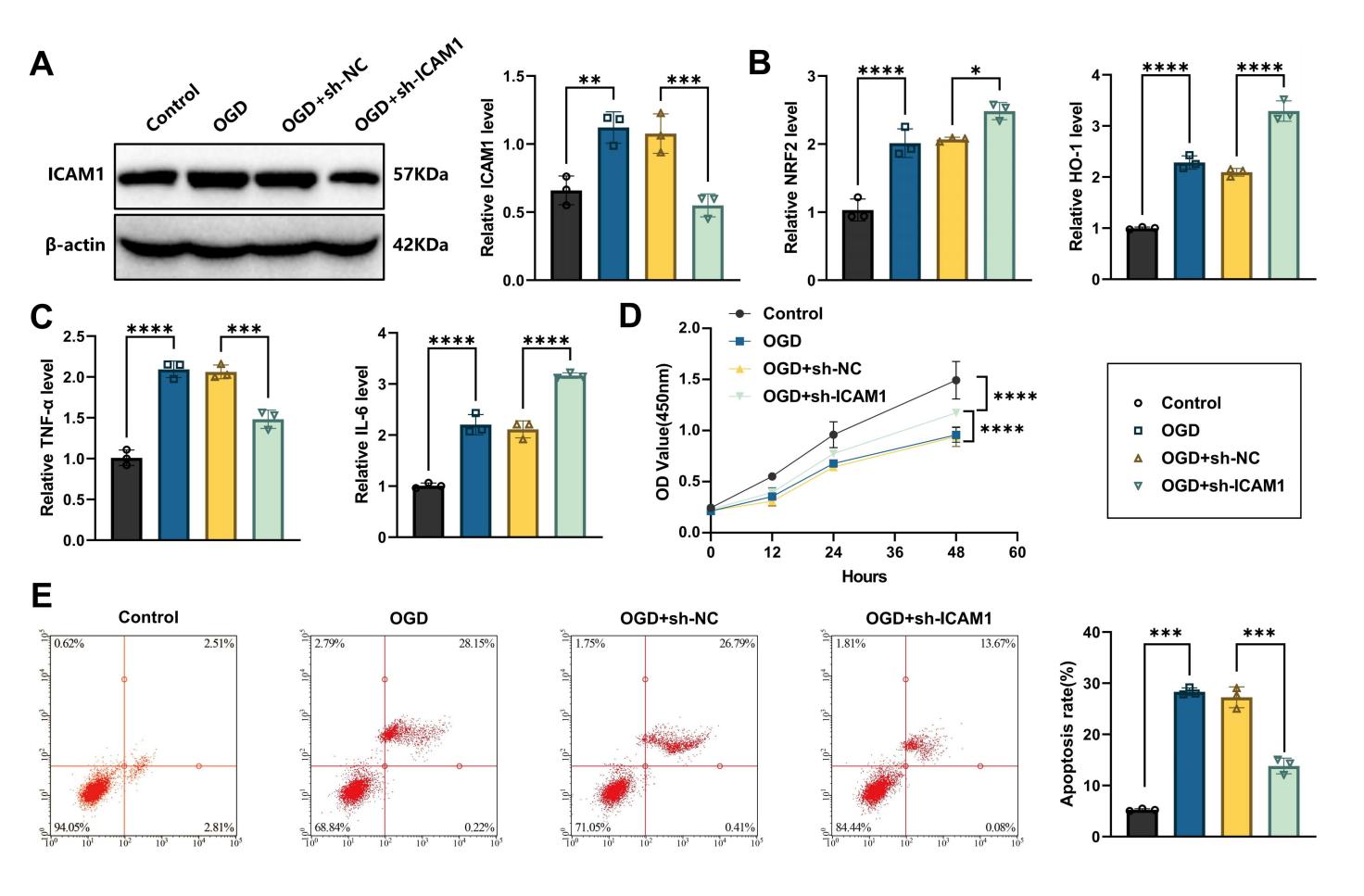
Figure S1. Effect of ICAM1 Knockdown on SH-SY5Y Cell Function.**

Note: (A) Western blot analysis of ICAM1 expression in SH-SY5Y cells; (B) RT-qPCR analysis of the expression levels of antioxidant stress-related genes (Nrf2 and HO-1) in SH-SY5Y cells; (C) RT-qPCR analysis of the expression levels of inflammatory factors (TNF-α and IL-6) in SH-SY5Y cells; (D) CCK-8 assay to assess SH-SY5Y cell proliferation; (E) Annexin V-FITC/PI flow cytometry to measure SH-SY5Y cell apoptosis rate. All data are presented as mean ± SEM. Experiments were repeated three times, and statistical analysis was performed using ANOVA followed by Tukey’s post-hoc test. **p* < 0.05, ***p* < 0.01, ****p* < 0.001, *****p* < 0.0001.

**Table S1. RT-qPCR Primer Sequence.**

| **Gene name** | **Primer Sequence** |
| --- | --- |
| NRF2 | Forward: 5’-AGGTTGCCCACATTCCCAAA-3’ |
|  | Reverse: 5’-AGTGACTGAAACGTAGCCGA-3’ |
| HMOX1 | Forward: 5’-AGGGAATTCTCTTGGCTGGC-3’ |
|  | Reverse: 5’-CTTCGCCCCCTCTGAAGTTT-3’ |
| TNF-α | Forward: 5’-CAAGGACAGCAGAGGACCAG-3’ |
|  | Reverse: 5’-TCCTTTCCAGGGGAGAGAGG-3’ |
| IL-6 | Forward: 5’-CCACCGGGAACGAAAGAGAA-3’ |
|  | Reverse: 5’-GAGAAGGCAACTGGACCGAA-3’ |
| β-actin | Forward: 5’-CCTTTGCCGATCCGCCG-3’ |
|  | Reverse: 5’-AATCCTTCTGACCCATGCCC-3’ |
